# Nitric oxide synthase 2A (NOS2A) polymorphisms are not associated with invasive pneumococcal disease

**DOI:** 10.1186/1471-2350-10-28

**Published:** 2009-03-23

**Authors:** Antony Payton, Debbie Payne, Limangeni A Mankhambo, Daniel L Banda, C Anthony Hart, William ER Ollier, Enitan D Carrol

**Affiliations:** 1 Centre for Integrated Genomic Medical Research, Stopford building, The University of Manchester, Oxford Road, Manchester, M13 9PT, UK; 2 Malawi-Liverpool-Wellcome Trust Clinical Research Programme, PO Box 30096, Blantyre, Malawi; 3 Division of Medical Microbiology, University of Liverpool, Daulby Street, Liverpool, L69 3GA, UK; 4 Division of Child Health, University of Liverpool, Alder Hey Children's NHS Foundation Trust,, Eaton Road, Liverpool, L12 2AP, UK

## Abstract

**Background:**

*Streptococcus pneumoniae *(pneumococcus) is responsible for over one million deaths per year, with young children, the elderly and immunocompromised individuals being most at risk. Approximately half of East African children have been reported to be asymptomatic carriers of pneumococcus with invasive infection occurring after the disruption of the respiratory membrane which is believed to be caused by the host immune response. Racial incidence of invasive pneumococcal disease (IPD) is higher in certain populations even after adjusting for environmental factors suggesting a genetic component to disease susceptibility. The nitric oxide synthase 2A (NOS2A) gene is responsible for the production of nitric oxide under pathological conditions including host defence against bacterial infection. Nitric oxide is a modulator of apoptotic and inflammatory cascades and endothelial permeability. We hypothesised that genetic variants within this gene may predispose to disease risk and survival.

**Methods:**

A cohort of 299 children with IPD (221 meningitis, 41 pneumonia and 37 with bacteraemia) and 931 age matched controls from Malawi were used in this study. We investigated nine haplotype tagging single nucleotide polymorphisms within the NOS2A gene and compared the presence or absence of the minor alleles in cases and controls and survivors and non-survivors within the cases.

**Results:**

We observed no significant associations between cases and controls or with survival in either all IPD cases or in the separate analysis of meningitis cases. A near significant association was obtained for the comparison of rs8078340 in cases and controls (p-value, 0.078). However, results were unadjusted for multiple testing.

**Conclusion:**

Our results suggest that polymorphic variation within the NOS2A gene does not influence invasive pneumococcal disease susceptibility or survival.

## Background

The pneumococcus is a principal contributor towards serious illness particularly in young children from developing countries. It colonises the mucosal surface of the upper respiratory tract and has been reported to have a nasopharyngeal carriage of 57%, 41% and 6% in East African individuals aged 0–4, 5–9 and 10–85 years, respectively [1]. Invasive infection is achieved by the disruption of the respiratory epithelial membrane which is believed to be initiated by the host's immune response in a Toll-like receptor 2 dependent manner [2,3]. This results in invasive pneumococcal disease (IPD) which includes pneumonia, meningitis and bacteraemia. Meningitis has the highest mortality rate (approximately 30%) with 21% of meningitis survivors suffering neurological deficits which include seizures, hearing loss and intellectual impairment [4]. HIV positive individuals have a higher incidence of disease with a reported 8.2 and 36.9-fold increase in adults and children, respectively [5]. Other factors such as overcrowding and exposure to smoke have also been associated with increased prevalence of IPD [6]. Disease severity is dependent upon a number of factors including age, co-morbid disease, pneumococcus strain, bacterial load and pneumolysin allelic variation [7]. Our group have shown that high blood and cerebral spinal fluid pneumococcal DNA loads are associated with a fatal outcome in Malawian children with IPD [8].

Certain racial groups including native American and Australian populations and African Americans have a higher incidence of IPD even after socio-economic factors have been adjusted for suggesting that genetic variation may predispose to disease development [9]. Current knowledge of genetic variation and immune response to IPD is limited although associations have been reported between polymorphisms within six genes of members of the family of inhibitors of nuclear factor (NF)-kappaB, NFKBIA and NFKBIE [10], the adaptor protein Mal [3], the Fc fragment of IgG, low affinity IIa, receptor (CD32) [11], interleukin-10 [12], C-reactive protein [13] and mannose-binding lectin (MBL) [14]. The only attempted replication of these findings has been with CD32 and MBL where subsequent investigations failed to find an association [15,16].

Nitric oxide (NO) is a pleiotropic substance that has been shown to regulate vascular permeability, motor control and host defence against bacterial and parasitic infections [17-19]. Under non-pathological conditions neuronal and endothelial nitric oxide synthase (NOS1 and NOS3, respectively) are responsible for NO production by catalysing the breakdown of L-arginine into NO and L-citrulline. Whilst, NOS1 and NOS3 are involved in the immune response, another isoform of NOS called inducible NOS (NOS2) is responsible for a much greater expression of NO in response to injury or infection [20]. NO is produced by the majority of immune response cells including alveolar macrophages which are the first cells involved in phagocytosis and early proinflammatory cytokine release during respiratory infection [21]. Successful phagocytosis of pneumococcus by macrophages has been shown to increase NOS2 and NO production [22]. This increase results in down-regulation of anti-apoptotic proteins, up-regulation of pro-apoptotic proteins, mitochondrial membrane permeabilisation and ultimately apoptosis of the macrophage. In the same study NOS2 inhibitors reduced bacterial killing and shifted macrophage death from apoptotic to necrotic. Apoptosis has been shown to play an important role in neuronal cell death caused by IPD and this process is believed to be responsible for the high mortality in meningitis [23]. NOS2 has also been shown to influence the inflammatory response to bacterial lipopolysaccharide through the regulation of the cytokine response [24]. Brain protein levels of IL-1β, IL-6, TNF-α, MIP-1α, MIP-2 are reduced in inducible NOS (iNOS) deficient, pneumococcal meningitis induced mice suggesting that iNOS derived NO plays a role in cytokine and chemokine upregulation [25]. This study also reported that iNOS-derived NO plays a role in peroxynitrite formation which disrupts the blood brain barrier. Finally, pneumolysin, a pore-forming hemolysin, and an important pneumococcal virulence factor has been shown to induce iNOS, and NO production in macrophages [26].

Here we investigate the influence of haplotype tagging SNPs (htSNPs) within the NOS2A gene (43.8 kb gene that codes for NOS2; chromosome position 17q11.2), on both disease susceptibility and survival in a cohort of children with IPD and age matched controls from Malawi.

## Methods

### Study population

The 299 children with IPD (aged 2 months to 16 years) recruited into this study were admitted from the Queen Elizabeth Central Hospital, Blantyre, Southern Malawi. HIV prevalence of paediatric inpatients in this area is 19% with 34% prevalence reported in children with bacterial meningitis [27,28]. Pneumococcal vaccine was not available and at time of admission only 2 participants were receiving antiretroviral therapy and 3 were on co-trimoxazole prophylaxis. Only children with confirmed IPD (by culture, PCR, microscopy or latex antigen tests) were included in the study. Pneumococcal pneumonia was confirmed by radiology and positive blood or lung aspirate by culture or PCR. Pneumococcal meningitis was confirmed by CSF cell count (>10 μl^-1^) and one of the following tests: CSF culture, gram stain, polysaccharide antigen and/or pneumococcal PCR positive. Of the 299 cases, 221 were diagnosed with meningitis, 41 with pneumonia and 37 with bacteraemia alone. Of the 41 cases with pneumococcal pneumonia, 13 (32%) were bacteraemic by blood culture or PCR and 31 (78%) were positive by PCR of lung aspirate. As the majority of patients with pneumococcal pneumonia do not have bacteraemia, we did not make this a requirement to fulfil the criteria of invasive pneumococcal disease. The number of cases who survived was 234 and the number who died was 65. The 931 controls consisted of age matched, afebrile and aparasitemic children from the same villages as the cases. HIV status was determined by either Unigold (Trinity Biotech, Wicklow, Ireland), Serocard (Trinity Biotech, Wicklow, Ireland) or Determine-HIV (Abbott Diagnostics Ltd, Maidenhead, U.K.) in children over 18 months and Amplicor HIV-1 Test version 1.5 (Roche Diagnostics, Indianapolis, U.S.A.) in those under 18 months. Further details on laboratory methods and management protocols can be found elsewhere [8]. Ethical approval was obtained from both The College of Medicine Research and Ethics Committee and the Liverpool School of Tropical Medicine Research and Ethics Committee.

### Genotyping

NOS2A polymorphism information was obtained from the HapMap  Yoruba population data. Haploview Version 4.1 was used to select htSNPs, with an r^2 ^threshold of 0.8 [29]. The htSNPs comprised: rs8078340, rs2779248, rs16966563, rs3794763, rs1137933, rs17718148, rs4796052, rs2297518, rs2314809, rs2297512. PCR reactions were carried out on PTC-225 Peltier Thermal Cyclers (MJ Research, Waltham, USA) in 384 well microtitre plates using 10 ng of genomic DNA with a final reaction volume of 10 μl. Primer details and PCR conditions can be obtained from the corresponding author. Five replication samples and two blank controls were used as quality controls for each plate. Genotyping was performed using Sequenom™ technology (San Diego, USA). All laboratory work was performed under the ISO 9001:2000 quality management requirements.

### Analysis

Hardy-Weinberg equilibrium was calculated using Golden Helixtree Genetics Analysis software version 5.0 (Golden Helix, Inc. MT, USA). The influence of genotype on cases and controls and survival in cases was performed in Stata v.8.2 (StataCorp, Texas, USA) using logistic regression analysis. The number of homozygous individuals for the minor allele was small (<50) for the majority of genotypes and they were pooled with heterozygous individuals. Analysis was therefore performed for the presence or absence of the minor allele. Data were adjusted for bacterial load, HIV status, disease type (pneumonia, meningitis or bacteraemia), age and gender for the analysis of genotype and survival. HIV status was not available for the controls and therefore case-control analysis was only adjusted for age and gender. Survival analysis was performed on all IPD cases and meningitis cases separately. Survival in pneumonia and bacteraemia cases was not performed due to the low number of deaths in each group (6 and 7, respectively).

## Results

Minor allele frequencies (MAF), Hardy-Weinberg equilibrium (HWE) p-values and successful genotype call rates are given in Table 1. SNP rs2297518 had a low call rate success (33%) and was excluded from analysis. MAF was compared against that given on HapMap for the Nigerian Yoruba in Ibadan (YRI) population. Five SNPs (rs2779248, rs16966563, rs1137933, rs4796052 and rs2297512) had similar frequencies (<3% difference). SNPs rs8078340, rs3794763, rs17718148, and rs2314809 had frequencies of 0.24, 0.27, 0.16, and 0.38 in the Malawi controls and 0.13, 0.11, 0.01, and 0.33 in the YRI population, respectively. MAF of our cases and controls were similar with the largest difference being 4% (rs8068149).

**Table 1:** NOS2A htSNP gene position, minor allele frequencies (MAF), successful genotype call rates and Hardy-Weinberg equilibrium p-values (HWE P) in Malawi IPD cases and controls

**Reference SNP**	**Position**	**Nucleotide substitution**	**MAF case**	**MAF control**	**Call rate**	**HWE P**
rs8078340	5' near gene	G>A	0.27	0.24	0.97	0.13
rs2779248	5' near gene	T>C	0.46	0.45	0.99	0.70
rs16966563	Exon 4 Pro68Pro	T>C	0.26	0.26	0.97	0.95
rs3794763	Intron 5	G>A	0.27	0.27	0.98	0.43
rs1137933	Exon 10 Asp385Asp	C>T	0.14	0.17	0.97	0.85
rs17718148	Intron 11	C>T	0.13	0.16	0.98	0.45
rs4796052	Intron 11	C>T	0.20	0.21	0.98	0.83
rs2297518	Exon 16 Leu608Ser	G>A	0.25	0.25	0.33	0.36
rs2314809	Intron 17	T>C	0.39	0.38	0.97	0.61
rs2297512	Intron 20	A>G	0.16	0.16	0.97	0.92

Association analysis results are given in Table 2. We observed no significant associations between cases and controls or with survival in either all IPD cases or in the separate analysis of meningitis cases. A near significant association was obtained for the comparison of rs8078340 in cases and controls (p-value, 0.078). However, results were unadjusted for multiple testing and the near significant association likely occurred by chance given we performed 27 comparisons.

**Table 2:** Comparison of NOS2A htSNP allele frequencies between cases and controls and survival in cases

**Reference SNP**	**Homozygou wild-type**		**Presence of minor allele**		**p-value**	**95% confidence interval for coefficient**
	n (%)	n (%)	n (%)	n (%)		
	
	Case-control					
	
	Cases	Controls	Cases	Controls		
	
rs8078340	172 (59)	485 (54)	122 (41)	412 (46)	0.078	-0.59, 0.03
rs2779248	260 (88)	797 (87)	37 (12)	121 (13)	0.431	-0.65, 0.28
rs16966563	203 (69)	634 (70)	90 (31)	270 (30)	0.714	-0.27, 0.39
rs3794763	193 (65)	575 (63)	102 (35)	337 (37)	0.331	-0.16, 0.48
rs1137933	183 (63)	580 (64)	109 (37)	326 (36)	0.908	-0.30, 0.34
rs17718148	212 (72)	677 (75)	81 (28)	229 (25)	0.718	-0.28, 0.40
rs4796052	201 (67)	673 (73)	97 (33)	240 (27)	0.106	-0.06, 0.59
rs2314809	158 (55)	485 (54)	131 (45)	415 (46)	0.956	-0.32, 0.30
rs2297512	157 (53)	496 (55)	138 (47)	407 (45)	0.913	-0.29, 0.32

	Survival (all IPD cases)					
	
	Non-survivors	Survivors	Non-survivors	Survivors		
	
rs8078340	33 (51)	139 (61)	32 (49)	90 (39)	0.675	-1.09, 0.70
rs2779248	57 (88)	203 (88)	8 (12)	29 (12)	0.936	-1.14, 1.24
rs16966563	41 (63)	162 (71)	24 (37)	66 (29)	0.935	-0.90, 0.98
rs3794763	39 (60)	154 (67)	26 (40)	76 (33)	0.101	-0.16, 1.75
rs1137933	36 (56)	147 (64)	28 (44)	81 (36)	0.592	-0.68, 1.19
rs17718148	40 (62)	172 (74)	25 (38)	59 (26)	0.811	-1.11, 0.86
rs4796052	40 (62)	161 (69)	25 (38)	72 (31)	0.194	-1.54, 0.31
rs2314809	37 (58)	121 (54)	27 (42)	104 (46)	0.684	-0.72, 1.10
rs2297512	37 (57)	121 (52)	28 (43)	110 (48)	0.558	-0.64, 1.19

	Survival (meningitis cases only)					
	
	Non-survivors	Survivors	Non-survivors	Survivors		
	
rs8078340	27 (47)	100 (62)	30 (53)	61 (38)	0.567	-1.20, 0.66
rs2779248	49 (86)	138 (85)	8 (14)	24 (15)	0.783	-1.37, 1.03
rs16966563	35 (61)	112 (70)	22 (39)	47 (30)	0.608	-0.93, 0.55
rs3794763	35 (61)	105 (65)	22 (39)	56 (35)	0.117	-0.20, 1.77
rs1137933	35 (63)	99 (62)	21 (37)	60 (38)	0.623	-0.72, 1.21
rs17718148	38 (67)	118 (73)	19 (33)	44 (27)	0.634	-1.25, 0.76
rs4796052	37 (65)	114 (70)	20 (35)	49 (30)	0.153	-1.66, 0.26
rs2314809	31 (55)	86 (54)	25 (45)	73 (46)	0.666	-0.75, 1.17
rs2297512	32 (56)	89 (55)	25 (44)	73 (45)	0.521	-0.64, 1.26

Data adjusted for age and sex

## Discussion

We have investigated nine htSNPs that span the NOS2A gene and found that they do not influence disease susceptibility or survival in a Malawi IPD cohort. These results suggest either NOS2A polymorphisms are not associated with the disease or that the genetic effect size was too small for our cohort of 299 cases and 931 controls to detect. This sample size gives us 85% power to detect an odds ratio of 1.5 for our cases and controls assuming a minor allele frequency of 0.25 (average of our htSNPs) and a significance level of 0.05. Subdividing our cases by survival reduces power to 82% for detecting an odds ratio of 2.4 using the same parameters. Given the high levels of NO that are produced in response to pneumococcal infection it may be reasonable to assume that a genetic effect would have to be large to impact on disease conversion or severity. However, sample size is a limiting factor for this study especially for the analysis of survival where we are comparing 65 non-survivors against 234 survivors.

Associations between NOS2A polymorphisms and other diseases have previously been reported. The exon 10 SNP rs1137933 has been associated with Crohns disease and the promoter SNP rs2779248 has been associated with ulcerative colitis [30]. In addition, an exon 16 SNP rs2297518 has been implicated in type-1 diabetes susceptibility, risk of non-Hodgkin lymphoma and atrophic gastritis [31-33]. Unfortunately, rs2297518 was excluded from our analysis due to a low genotype success. This non-synonymous SNP (Leu608Ser) cannot therefore be excluded from association with IPD. The largest study of NOS2A variation in multiple sclerosis demonstrated that common variation within NOS2A alone does not appear to significantly influence disease severity [34].

## Conclusion

Whilst we cannot rule out that NOS2A influences disease susceptibility or severity to IPD, our results suggest the impact if any will be small. However, evidence from other studies suggest that the immune response is important both in the conversion of the asymptomatic carriage to causing invasive disease and in the severity of the disease. Other immune response genes should therefore be the focus of future IPD genetic association studies.

## Abbreviations

NOS2A: nitric oxide synthase 2A; IPD: invasive pneumococcal disease; NO: Nitric oxide; iNOS: inducible nitric oxide synthase.

## Competing interests

The authors declare that they have no competing interests.

## Authors' contributions

AP and DP carried out the genotyping. AP performed the statistical analysis AP and EDC drafted the manuscript. EDC, LAM and DLB recruited the patients and controls. EDC, CAH and WERO participated in the design of the study. All authors read and approved the final manuscript.

## Pre-publication history

The pre-publication history for this paper can be accessed here:


